# Application of Ultrasonography and Radiography in Detection of Hemothorax; a Systematic Review and Meta-Analysis 

**Published:** 2016

**Authors:** Vafa Rahimi-Movaghar, Mahmoud Yousefifard, Parisa Ghelichkhani, Masoud Baikpour, Abbas Tafakhori, Hadi Asady, Gholamreza Faridaalaee, Mostafa Hosseini, Saeed Safari

**Affiliations:** 1 Sina Trauma and Surgery Research Center, Tehran University Medical Sciences, Tehran, Iran.; 2 Department of Physiology, School of Medicine, Tehran University of Medical Sciences, Tehran, Iran.; 3 Department of Intensive Care Nursing, School of Nursing and Midwifery, Tehran University of Medical Sciences, Tehran, Iran.; 4 Department of Medicine, School of Medicine, Tehran University of Medical Sciences, Tehran, Iran.; 5 Department of Neurology, School of Medicine, Imam Khomeini Hospital, Tehran University of Medical Sciences, Tehran, Iran.; 6 Iranian Center of Neurological Research, Tehran University of Medical Sciences, Tehran, Iran.; 7 Department of Occupational Health Engineering, Faculty of Public Health, Tehran University of Medical Sciences, Tehran, Iran.; 8 Department of Emergency Medicine, Maragheh University of Medical Sciences, Maragheh, Iran.; 9 Department of Epidemiology and Biostatistics, school of Public Health, Tehran University of Medical Sciences, Tehran, Iran.; 10 Department of Emergency Medicine, Shohedaye Tajrish Hospital, Shahid Beheshti University of Medical Sciences, Tehran, Iran.

**Keywords:** Hemothorax, ultrasonography, radiography, diagnostic tests, routine

## Abstract

**Introduction:**

Hemothorax is one of the most prevalent injuries caused by thoracic traumas. Early detection and treatment of this injury is of utmost importance in prognosis of the patient, but there are still controversial debates on the diagnostic value of imaging techniques in detection of hemothorax. Therefore, the present study aimed to evaluate the diagnostic value of chest ultrasonography and radiography in detection of hemothorax through a systematic review and meta-analysis.

**Methods:**

Two independent reviewers performed an extended systematic search in databases of Medline, EMBASE, ISI Web of Knowledge, Scopus, Cochrane Library, and ProQuest. Data were extract and quality of the relevant studies were assessed. The number of true positive, false positive, true negative and false negative cases were extracted and screening performance characteristics of two imaging techniques were calculated using a mixed-effects binary regression model.

**Results:**

Data from 12 studies were extracted and included in the meta-analysis (7361 patients, 77.1% male). Pooled sensitivity and specificity of ultrasonography in detection of hemothorax were 0.67 (95% CI: 0.41-0.86; I2= 68.38, p<0.001) and 0.99 (95% CI: 0.95-1.0; I2= 88.16, p<0.001), respectively. These measures for radiography were 0.54 (95% CI: 0.33-0.75; I2= 92.85, p<0.001) and 0.99 (95% CI: 0.94-1.0; I2= 99.22, p<0.001), respectively. Subgroup analysis found operator of the ultrasonography device, frequency of the transducer and sample size to be important sources of heterogeneity of included studies.

**Conclusion:**

The results of this study showed that although the sensitivity of ultrasonography in detection of hemothorax is relatively higher than radiography, but it is still at a moderate level (0.67%). The specificity of both imaging modalities were found to be at an excellent level in this regard. The screening characteristics of ultrasonography was found to be influenced of the operator and frequency of transducer

## Introduction:

Chest traumas are one of the most important causes of mortality in the fourth decade of life (1, 2). 25% of trauma mortalities are due to these injuries (3). In this regard, imaging techniques play a vital role in management of these patients. Although some thoracic traumas are treated according to clinical findings of the patient before performing any imaging studies, but in many cases application of various imaging modalities such as computed tomography (CT) scan, plain chest X-ray (CXR) and ultrasonography are necessary. Among these modalities, CT scan is the gold standard for identification of intra thoracic injuries following trauma with a significantly high diagnostic value for occult and soft tissue injuries (4-9). However, limited availability of CT scan in all medical centers, limitations in patient transfer to radiology department and radiation exposure led the researchers to look for other diagnostic tools (10). 

CXR is the first diagnostic test for screening of thoracic traumas but the limitations of supine radiography in some traumatic injuries such as pneumothorax is confirmed in various studies (11, 12). Moreover, Low diagnostic yield of routine chest radiography in patients with thoracic injuries encouraged the researchers to search for alternative imaging techniques (11-14). Accordingly, in recent years scoring systems such as thoracic injury rule out criteria (TIRC) and national emergency X-Radiography utilization study (NEXUS) have been developed to lower the burden of unnecessary imaging studies (15, 16).

**Figure 1:**
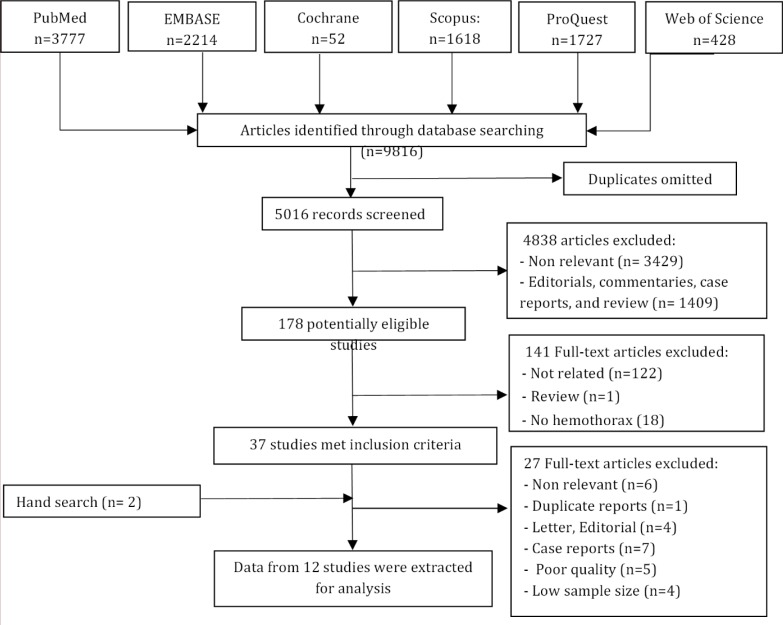
Flowchart of the study

Major attention has recently been drawn to ultrasonography as a quick screening tool with minimum complications (17). It has shown to have superior diagnostic value in detection of thoracic traumatic injuries compared to chest radiography (18-22). However, diagnostic accuracy of ultrasonography is highly dependent on the skills of the operator and is usually not reliable in detection of injuries without bleeding or free fluid (23-25).

Hemothorax is one the traumatic thoracic injuries caused by accumulation of blood in pleural cavity. This lesion along with pneumothorax is present in 83% of thoracic traumas (26). However, detection of this complication via chest radiography is not possible unless the volume of hemothorax exceeds 175 milliliters (27). Moreover, the diagnostic value of ultrasonography for hemothorax is still a matter of debate as well (18, 19, 28, 29). Recently multiple systematic reviews have been published to evaluate the diagnostic value of ultrasonography and chest radiography in detection of thoracic traumas, but almost all of them have assessed pneumothorax. These reviews showed a higher sensitivity of ultrasonography in identification of pneumothorax compared to chest radiography (29, 30). None of these surveys has taken a meta-analytic approach towards assessing diagnostic value of these imaging modalities in detection of hemothorax. Therefore, the present study aimed to evaluate the diagnostic accuracy of chest ultrasonography and radiography in detection of hemothorax through a systematic review of available literature and meta-analysis.

**Table 1:** Charecteristics of included studies

**Study **	**No. of patient** **(+ / -)** ^1^	**Age** ^2^ ** (years)**	**Male (%)**	**Reference / Index**	**Transducer / Operator**	**Sampling**	**Weaknesses**
**Ma 1997 (** 27 **)**	26 / 214	NR	NR	CT / US, CXR	3.5-to 2.5-MHz / EP	Consecutive	Retrospective design
**Abboud 2003 (** 28 **)**	14 / 126	38 (5–89)	NR	CT / US	3.75 MHz / EP	Convenience	Time interval between US and CT scan was varied; Blinding was not performed
**Brooks 2004 (** 18 **)**	12 / 49	NR	NR	CT / US	4- to 2-MHz / EP	Convenience	Small sample size; Possibility of selection bias
**Traub 2007 (** 31 **)**	16 / 125	47 (18-89)	75	CT / CXR	NA / Radiologist	Convenience	Retrospective design Possibility of selection bias
**Hyacinthe 2012 (** 19 **)**	35 / 202	39 (22-51)	82	CT / US	5- to 2-MHz / EP	Consecutive	Possibility of selection bias
**Poveda 2012 (** 32 **)**	47 / 21	39 (16-70)	89.7	Surgery / US	3.75 MHZ / Radiologist	Convenience	Possibility of selection bias
**Błasińska 2013 (** 33 **)**	24 / 36	NR	NR	CT / CXR	NA / Radiologist	Consecutive	Low sample size
**Uz 2013 (** 34 **)**	21 / 86	37 (19.8)	80.4	CT/ US	10-to 5-MHz / Radiologist	Consecutive	Low sample size
**Chardoli 2013 (** 35 **) **	14 / 186	38 (16-90)	84	CT / CXR	NA / EP	Convenience	The interpretation of the CXR and CT were not in blind fashion Possible selection bias
**Leblanc 2014 (** 36 **)**	19 / 26	36 (15-56)	71	CT/ US, CXR	5-to 1-MHz / Intensivist	Convenience	Low sample size Possibility of selection bias
**Langdorf 2015 (** 37 **)**	230 / 5682	≥ 15	57.4	CT/ CXR	NA / Radiologist	Convenience	Possibility of selection bias Variation in timing of CT and CXR
**Vafaei 2015 (** 38 **)**	29 / 123	31 (4-67)	77.6	CT / US, CXR	3.5-to 7-MHz / EP	Convenience	Possibility of selection bias

1, (+ / -): Number of patient with hemothorax / number of patient without hemothorax; 2, Number are presented as mean ± standard deviation or (range). CT: Computed tomography; CXR: Chest radiography; EP: Emergency physician; NA: Not applicable; NR: Not Reported; US: Ultrasonography

## Methods:

***Search strategy and selection criteria***

Two independent reviewer (M.Y, P.G) performed an extended systematic search in databases of Medline (via PubMed), EMBASE, ISI Web of Knowledge, Scopus, Cochrane Library, and ProQuest. We screened google scholar for further studies. The objective was to find surveys evaluating the diagnostic accuracy of ultrasonography or chest radiography in detection of hemothorax. 

Keywords were chosen according to Medical Subject Heading (MeSH) terms and EMTREE. Similar keywords were used for search in other databases. These keywords were terms related to ultrasonography and radiography including “Ultrasonography” OR “Sonography” OR “Ultrasound” OR “Chest Film” OR “Chest Radiograph” combined with hemothorax related terms including “Hemothorax” OR “Haemothorax” OR “Haemorrhagic Pleural Effusion”. In order to find further studies or unpublished surveys, hand-search was performed on the list of bibliography of relevant studies and the authors were contacted in cases where the data could not be extracted from the survey. 

Only the original articles and the surveys conducted on human subjects were included. Review and editorial articles, case reports, letters to editors, poster presentations, and meeting abstracts were excluded. Studies were included only if they presented an evaluation of the diagnostic value of ultrasonography or chest radiography in hemothorax detection. Other inclusion criteria were as follows: confirmation of injury via CT scan or surgery, performance of radiography or ultrasonography for all patients, presentation of true positive, true negative, false positive, and false negative cases (in the article, through contacting the authors, or using web-based calculators). No time or language limitations were applied. Both retrospective and prospective studies were included. 

***Data extraction***

Two of the authors (M.Y, P.G) independently worked on summarizing the data including assessment of quality of studies, information related to the subjects (age, gender, the number of patients with/without hemothorax, the etiology of hemothorax), the characteristics of ultrasonography device (transducer, frequency), operators and the physicians in charge of interpreting the imaging, blinding status, sampling method (consecutive, convenience), study design (retrospective, prospective), reference test, and the number of true positive, false positive, true negative, and false negative cases. Disagreements were discussed with the third author (M.H) and a solution was proposed. In cases of data inaccessibility, the corresponding authors of the articles were contacted. Data presented as charts were extracted via the method proposed by Sistrom and Mergo (39). In cases where only the sensitivity and specificity were presented in the article, reliable web-based programs were used to calculate the number of true positive, false positive, true negative and false negative cases. 

Quality of the studies were evaluated according to the Quality Assessment of Diagnostic Accuracy Studies (QUADAS2) guideline (40). Assessment was performed based on the criteria established for designing a diagnostic survey considering various biases including selection, performance, recording and reporting bias. 

***Statistical analysis***

Analysis was performed via STATA 11.0 statistical software through MIDAS module. The number of false positive, false negative, true positive and true negative cases were recorded. Then, pooled sensitivity, specificity, positive likelihood ratio, and negative likelihood ratio of chest ultrasonography and radiography in detection of hemothorax were calculated with 95% confidence interval (95% CI). In cases of data presented for each hemi-thorax separately, we also included the information, separately. In the present study, mixed-effects binary regression model, a type of random effect model, was used because of the presence of significant heterogeneity between the studies. Heterogeneity was assessed through application of I2 and χ2 tests. A p value of less than 0.1 along with an I2 greater than 50% were considered as positive heterogeneity (41).

To identify the source of heterogeneity, subgroup analysis was carried out using a bivariate mixed-effects binary regression model. Subgroup analyses were performed according to sampling method (consecutive / convenience), operator of the ultrasonography device (emergency physician/ other specialists) or the interpreting physician of CXR, the frequency of ultrasonography transducer (1-5 MHz/ 5-10 MHz) and sample size (less than 100 patients/ more than 100 patients).

## Results:

***Study characteristics***

**Figure 2:**
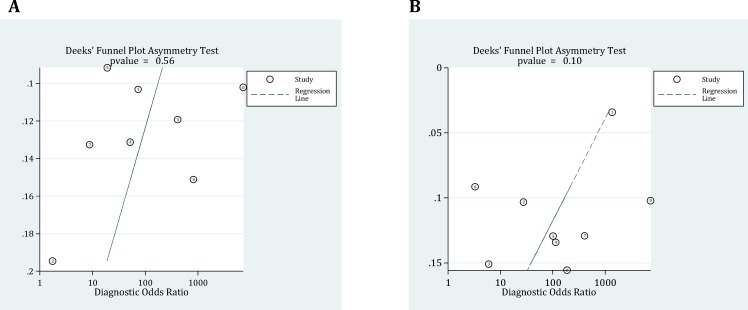
Deeks’ funnel plot asymmetry test for assessment of publication bias. P values < 0.05 were considered as significant. Ultrasonography (A); Radiography (B). ESS: Effective sample sizes

In literature review, 178 potentially relevant studies were identified, of which 37 met the inclusion criteria. Eventually 12 surveys were included in final meta-analysis (18, 19, 27, 28, 31-38) (Figure 1). Data on 7361 trauma patients including 487 with hemothorax and 6874 without were extracted (77.1% male). Table 1 summarizes the baseline characteristics of included studies. Diagnostic accuracy of ultrasonography and radiography were evaluated simultaneously in three studies (27, 36, 38), whereas the accuracy of ultrasonography and radiography were assessed individually in five (18, 19, 28, 32, 34) and four (31, 33, 35, 37) surveys, respectively. Significant heterogeneity was observed between the studies (P<0.001). No publication bias was found (Figure 2).

***Meta-analysis***

**- Ultrasonography**

**Figure 3:**
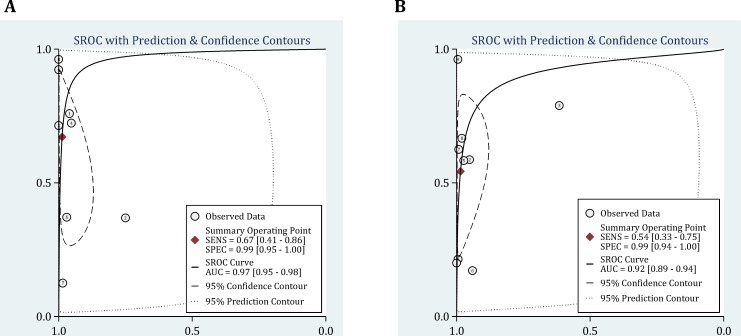
Summary receiver operative curves (SROC) for ultrasound (A) and chest radiography (B) in detection of hemothorax. AUC: Area under the curve; SENS: Sensitivity; SPEC: Specificity

**Figure 4:**
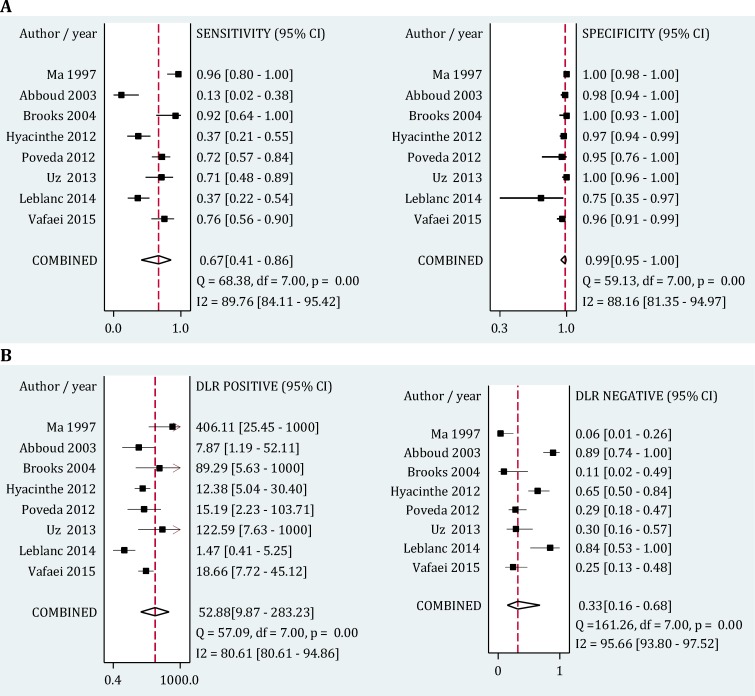
Forest plot of screening performance characteristics of chest ultrasonography in detection of hemothorax. Sensitivity and specificity (A); Diagnostic likelihood ratio (DLR) (B). CI: Confidence interval

Area under the curve of summary Receiver Operative Curves (SROC) for ultrasonography in detection of hemothorax was 0.97 (95% CI, 0.95-0.98) (Figure 3-A). Its pooled sensitivity and specificity in detection of hemothorax were 0.67 (95% CI: 0.41-0.86; I2= 68.38, p<0.001) and 0.99 (95% CI: 0.95-1.0; I2= 88.16, p<0.001), respectively. In addition, positive and negative likelihood ratios were computed to be 52.88 (95% CI: 9.87-283.23; I2= 80.61, p<0.001) and 0.33 (95% CI: 0.16-0.68; I2= 95.66, p<0.001), respectively (Figure 4).

Subgroup analysis found sampling method (consecutive/ convenience), operator (emergency physician/ other specialists), frequency of the transducer (1-5 MHz/ 5-10 MHz), and sample size (less than 100 patients/ more than 100 patients) to be important sources of heterogeneity among studies. Sensitivity of the surveys with consecutive sampling methods were significantly higher than the other studies (0.76 vs. 0.61), but their specificity did not differ considerably (1.0 vs. 0.97). Moreover the sensitivity of ultrasonography in detection of hemothorax was found to be significantly higher when the procedure was performed via an emergency physician (0.70 vs. 0.62) or using a 5-10 MHz transducer (0.75 vs. 0.64) (Table 2).

**- Radiography**

**Figure 5:**
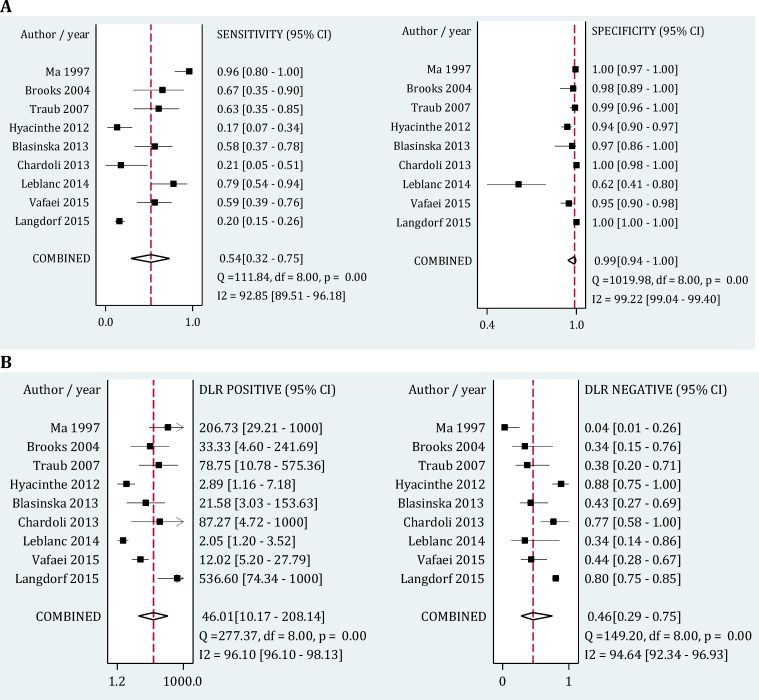
Forest plot of screening performance characteristics of chest radiography in detection of hemothorax. Sensitivity and specificity (A); Diagnostic likelihood ratio (DLR) (B). CI: Confidence interval

As presented in Figure 3-B, area under the SROC curve for radiography was 0.92 (95% CI: 0.89-0.94). Pooled sensitivity and specificity of this modality in detection of hemothorax were 0.54 (95% CI: 0.33-0.75; I2= 92.85, p<0.001) and 0.99 (95% CI: 0.94-1.0; I2= 99.22, p<0.001), respectively. Its positive and negative likelihood ratios were also 46.01 (95% CI: 10.17-208.14; I2= 96.10, p<0.001) and 0.46 (95% CI: 0.29-0.75; I2= 95.66, p<0.001), respectively (Figure 5). 

Subgroup analysis showed that sampling method (consecutive / convenience), the interpreting physician (emergency physician/ other specialists), and sample size (less than 100 patients/ more than 100 patients) were important sources of heterogeneity between the studies. Sensitivity of radiography was significantly higher in surveys with consecutive sampling methods (0.61 vs. 0.51) and with sample sizes of less than 100 patients (0.69 vs.0.46).

## Discussion:

Sonography, as one of the most available screening tools in emergency settings, is useful for various clinical applications but its diagnostic value in traumatic thoracic injuries is still a controversial subject. The present study is the first to conduct a systematic review with meta-analytic approach on one of the most important thoracic traumas. The results of this study illustrated the relatively higher sensitivity of ultrasonography in this regard, but it is still at a moderate level. The specificity and positive likelihood ratios calculated for both of these modalities were same and excellent. 

The results of subgroup analysis showed that the sensitivity of ultrasonography was influenced by the operator of the ultrasound device and frequency of transducer but the specificity of this modality is not affected by them. Accordingly, as ultrasonography performed by an emergency physician has a higher diagnostic value compared to other physicians. This finding might be due to awareness of the emergency physician about the clinical condition of the patient. Although in 8 studies the operators were blinded and in the other 4 the setting was not mentioned, a complete unawareness of the emergency physician about the patients’ clinical condition seems unlikely. Since these physicians are the first line of the medical team responsible for treatment of trauma patients, based on their experience they might suspect the presence of hemothorax according to the history and clinical findings of the subjects and consequently pay much more attention to find sonographic evidence of this complication. The diagnostic value of chest radiography in detection of hemothorax is neither affected by the operator nor by the interpreting physician, since the physician or the radiologist is not in direct contact with the patients when interpreting their radiographs.

Ebrahimi et al. (29) found no significant relation between frequency of transducer and detection of pneumothorax but the present survey yielded opposite results regarding detection of hemothorax. This might be due to the fact that the sound wave emitted from the transducer easily moves through fluids (high penetrating power in fluids), since the amount of energy absorbed by the fluids is very low. Therefore, ultrasonography with higher frequencies is able to produce clearer images with higher resolutions (42), an event that does not occur in pneumothorax because propagation of the sound wave through the air is associated with loss of energy and so pictures with higher resolutions are not necessarily yielded with higher frequencies. 

The minimum amount of fluid that can be detected by each of these modalities is different, 175 milliliters for radiography and only 20 milliliters for ultrasonography (27).

According to subgroup analysis, diagnostic accuracy of radiography in detection of hemothorax is influenced by the sample size of the survey. The results showed that in the studies with sample sizes of less than 100 patients, the sensitivity of radiography was reported to be higher. This finding could be due to probable selection bias in studies with smaller sample sizes, which might have led to evaluation of patients with greater volumes of free fluids that increases their chance of detection via radiography (43). 

**- Limitations**

Since all included studies were observational precise evaluation of causal relationships was not possible. The skills of the operator in performing ultrasonography were not considered in any of these surveys and the effect of this bias is not clear in the present study. Finally a significant heterogeneity was found between the surveys whose effects were tried to be minimized by application of mixed random effect model and subgroup analysis. 

**Table 2:** Subgroup analysis of diagnostic accuracy for chest radiography and ultrasonography in detection of hemothorax

**Covariate**	**No. of studies**	**Bivariate random-effect model**					
		**Sensitivity (95% CI)**	**P**	**Specificity (95% CI)**	**p**	**Heterogeneity, I** ^2^	**P** *
**Ultrasonography**							
**Patient enrollment**							
Consecutive	3	0.76 (0.45-1.00)	0.56	1.00 (0.99-1.00)	0.86	10.00 %	0.33
Convenience	5	0.61 (0.31-0.92)		0.97 (0.93-1.00)			
**Operator**							
Emergency physi cian	5	0.70 (0.42-0.99)	0.68	0.99 (0.98-1.00)	0.02	0.00 %	0.59
Other physician	3	0.62 (0.23-1.00)		0.97 (0.90-1.00)			
**Sample size**							
< 100	5	0.70 (0.41-0.98)	0.72	0.99 (0.97-1.00)	0.08	0.00 %	0.74
≥ 100	3	0.63 (0.24-1.00)		0.98 (0.95-1.00)			
**Frequency of transducer**							
1-5 MHz	5	0.64 (0.37-0.92)	0.55	0.99 (0.97-1.00)	0.12	0.00 %	0.94
5-10 MHz	3	0.75 (0.37-1.00)		0.99 (0.95-1.00)			
**Radiography**							
**Patient enrollment**							
Consecutive	3	0.61 (0.24-0.98)	0.65	0.98 (0.94-1.00)	0.03	0.00 %	0.88
Convenience	6	0.51 (0.23-0.78)		0.99 (0.97-1.00)			
**Operator**							
Emergency physi cian	5	0.54 (0.24-0.84)	0.96	0.99 (0.97-1.00)	0.07	0.00 %	0.99
Other physician	4	0.55 (0.22-0.87)		0.99 (0.96-1.00)			
**Sample size**							
<100	3	0.69 (0.38-1.00)	0.32	0.94 (0.81-1.00)	0.38	0.00 %	0.99
≥ 100	6	0.46 (0.21-0.72)		0.99 (0.99-1.00)			

* , P value < 0.1 was considered as significant for heterogeneity; CI: Confidence interval.

## Conclusion:

The results of this study showed that although the sensitivity of ultrasonography in detection of hemothorax is relatively higher than radiography, but it is still at a moderate level (0.67%). The specificity of both imaging modalities were found to be at an excellent level in this regard. The screening characteristics of ultrasonography was found to be influenced of the operator and frequency of transducer.
